# Syngnathia and associated congenital anomalies in an infant: A case report

**DOI:** 10.1016/j.ijscr.2025.111773

**Published:** 2025-08-06

**Authors:** Farnoosh Mohammadi, Zahra Sadat Modarresi, Saeed Hasani Mehraban, Rahele Mousavi

**Affiliations:** a Department of Oral and Maxillofacial Surgery, Dental School, Tehran University of Medical Sciences, Tehran, Iran

**Keywords:** Congenital syngnathia, Craniofacial anomalies, Xp22.31 deletion, Surgical intervention, Case report

## Abstract

**Introduction:**

Congenital syngnathia, a rare craniofacial anomaly characterized by maxillomandibular fusion, has challenges in feeding, respiration, and development. While often associated with syndromes or genetic mutations, its etiology remains unclear. Here, we presented syngnathia in a preterm infant with additional anomalies, such as hemangiomas and cardiac defects.

**Presentation of case:**

A 15-week preterm female presented with syngnathia (mucosal and bony fusion), microcephaly, thigh hemangioma, and cardiac defects (patent ductus arteriosus/ventricular septal defect). Genetic testing revealed a 1.66 Mb Xp22.31 deletion. Surgical separation with custom stent placement restored oral function, with no recurrence at follow-up. Postoperative stability and improved feeding were achieved.

**Discussion:**

There is a need for early surgical intervention and multidisciplinary care in managing congenital syngnathia, especially when associated with additional anomalies such as microcephaly, hemangiomas, and cardiac defects. The case highlights potential syndromic associations, the utility of imaging and genetic evaluation, and the role of tailored postoperative strategies.

**Conclusions:**

Early surgical intervention, multidisciplinary care, and genetic evaluation are necessary in syngnathia management. The Xp22.31 deletion expands the phenotypic spectrum, though syndromic links remain unclear. Custom stents and long-term monitoring optimize outcomes.

## Highlights

• Early surgery and stenting restored oral function in congenital syngnathia.
• Case links syngnathia with Xp22.31 deletion and multiple anomalies.
• Custom stent use prevented refusion after bony adhesion separation.
• Imaging and genetics are key in diagnosing complex craniofacial anomalies.
• Multidisciplinary care improves outcomes in rare craniofacial conditions.

## 1 Introduction

Syngnathia refers to the congenital fusion of the maxilla and mandible, which can be either fibrous, bony, or a combination of both. This rare craniofacial disorder, which was initially reported in 1936, can range from mild mucosal adhesion (i.e., synechiae) to complete bony fusion (i.e., synostosis) [1]. It can lead to severe difficulty with mouth opening, feeding, and respiration [1,2]. The condition is often associated with other craniofacial anomalies, including cleft lip, cleft palate, and several tongue and jaw malformations, which cleft palate is the most frequent one [2]. Syngnathia is frequently observed in syndromes such as Van der Woude, popliteal pterygium, and craniofacial microsomia [2]. The exact etiology is still unclear, however genetic factors, including mutations in the *FOXC1* and *FGF8* genes, are suggested that can play a role in the severity of the condition, while environmental causes, such as teratogenic exposure and abnormal vascularization, have also been implicated [1,3]. Moreover, disturbances in craniofacial development, such as failure in neural crest cell migration and inappropriate separation of the first pharyngeal arch, may contribute to its pathogenesis [3].

The diagnosis is based on clinical and radiological examination, with cases classified into two types: type 1 (soft tissue fusion) and type 2 (bony fusion) [4]. Recently, diagnostic tools like ultrasound and MRI for detecting conditions such as micrognathia, polyhydramnios, and mouth closure status have been used [3]. Furthermore, diagnostic methods typically involve 3D facial scans for detailed visualization of the fusion sites, which is important for surgical interventions planning [5].

Management of congenital syngnathia involves both pre-surgical and surgical considerations. Feeding difficulties are a challenge due to restricted mouth opening, with nasogastric tube or parenteral feeding might be necessary. The surgical approach varies, but the transoral method is preferred for both types of syngnathia, although extraoral approaches are occasionally required, particularly for type 2 cases. Post-surgical outcomes are generally favorable, with the majority of patients achieving a functional mouth opening, while recurrence is more common in type 2 syngnathia. Airway management during surgery often involves nasotracheal intubation or tracheostomy. Moreover, post-surgical jaw exercises are critical in maintaining jaw mobility, and survival rates remain high despite some cases experiencing recurrence or complications [6].

The condition is rare, and as such, there are few previous case reports on this disease which determined several presentations and management strategies [1,4,7,8]. While surgical interventions have shown improvements in managing the condition, there is still uncertainty regarding the best approaches to the diagnosis and treatment. Moreover, the rarity of syngnathia highlight the importance of reporting each case to provide suggestions for these patients. In this case report, we present syngnathia in a preterm infant with additional anomalies, including hemangiomas and cardiac defects. Our report aims to highlight the importance of surgical intervention for syngnathia and provide insight into the management of such a complex case.

## 2 Case presentation

The work has been reported in line with the SCARE criteria [9]. A 15-week female infant, born preterm at 34 weeks of gestation via cesarean section due to fetal distress, was referred to the neonatal intensive care unit (NICU) for evaluation of multiple congenital anomalies. She was the first child of a non-consanguineous Iranian couple. The pregnancy was not complicated by polyhydramnios and there was no history of teratogenic exposure or relevant familial genetic disorders.

**Fig. 1:**
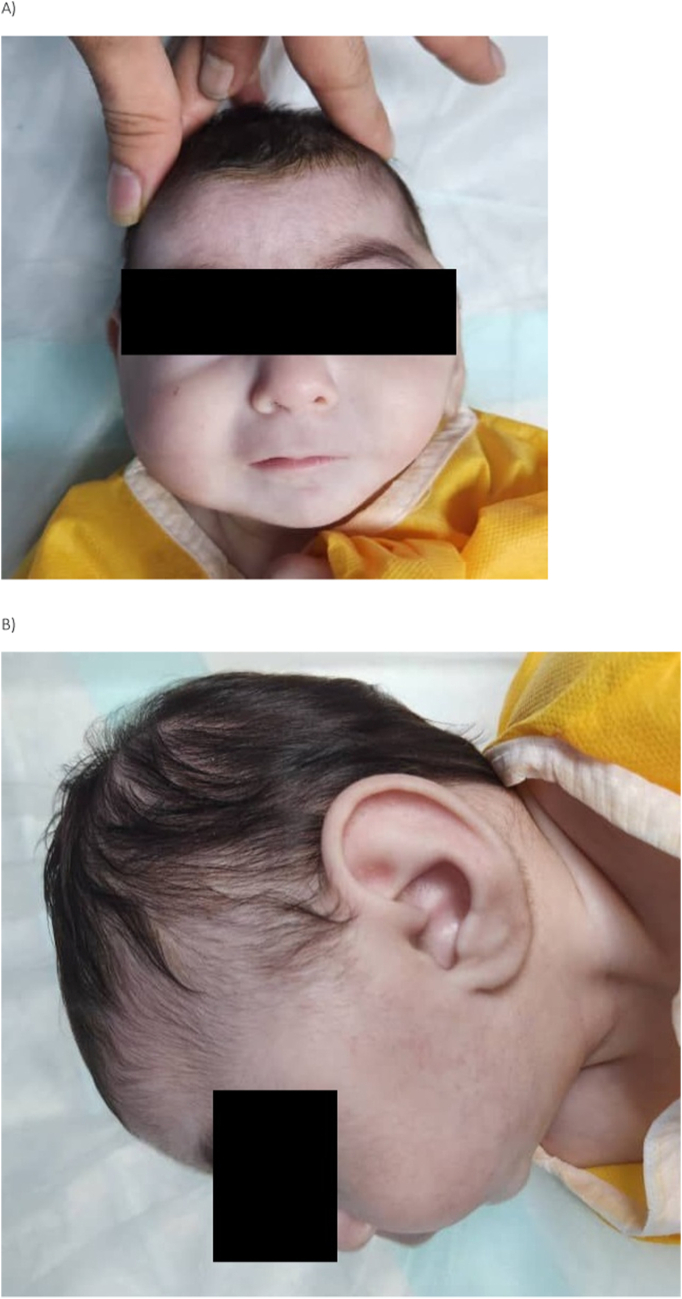
Clinical features of microcephaly in the case. (A) frontal view, (B) lateral view.

**Fig. 2:**
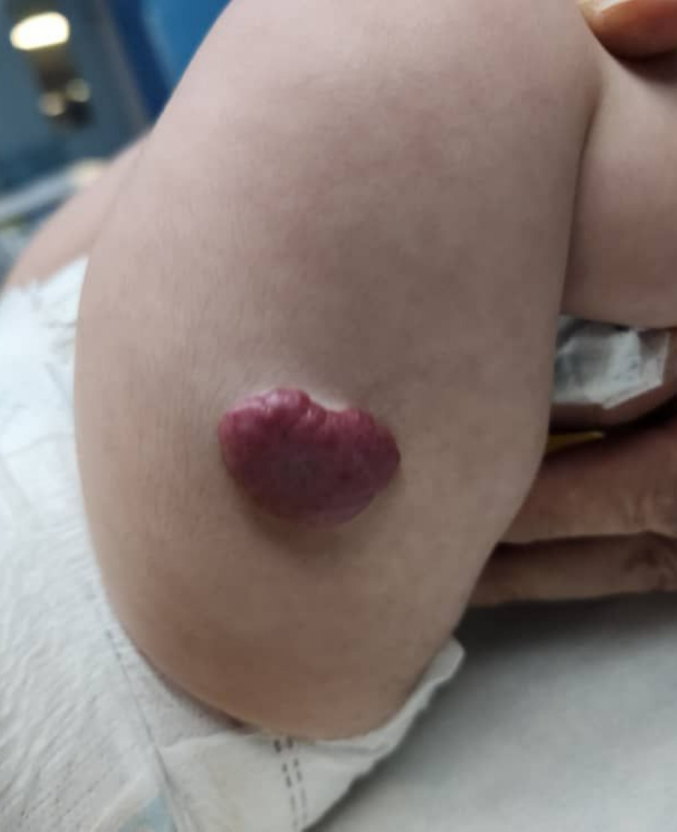
Clinical photograph showing a distinct hemangioma located on the right thigh of the patient. The lesion is characterized by its well-demarcated borders and raised appearance.

At birth, the infant weighed 2500 g, with a head circumference (HC) of 35 cm and a biparietal head circumference (BHC) of 30 cm. She was noted to have craniofacial dysmorphism, including microcephaly (Fig. 1), fusion of the maxilla and mandible (syngnathia), and a hemangioma on the right thigh measuring 2.5 × 2.0 cm (Fig. 2). Echocardiography on the first day of life revealed a small patent ductus arteriosus (PDA) and a small ventricular septal defect (VSD).

## 3 Clinical findings

Physical examination revealed restricted oral opening due to both mucosal and bony fusion of the maxilla and mandible, consistent with syngnathia. No cleft lip or palate was observed. The hemangioma on the right thigh was non-tender, with no ulceration or evidence of regression. Neurological examination did not demonstrate hypotonia, and cranial nerve function appeared intact. Cardiovascular findings showed a grade II/VI systolic murmur without hemodynamic compromise.

## 4 Diagnostic assessment

**Fig. 3:**
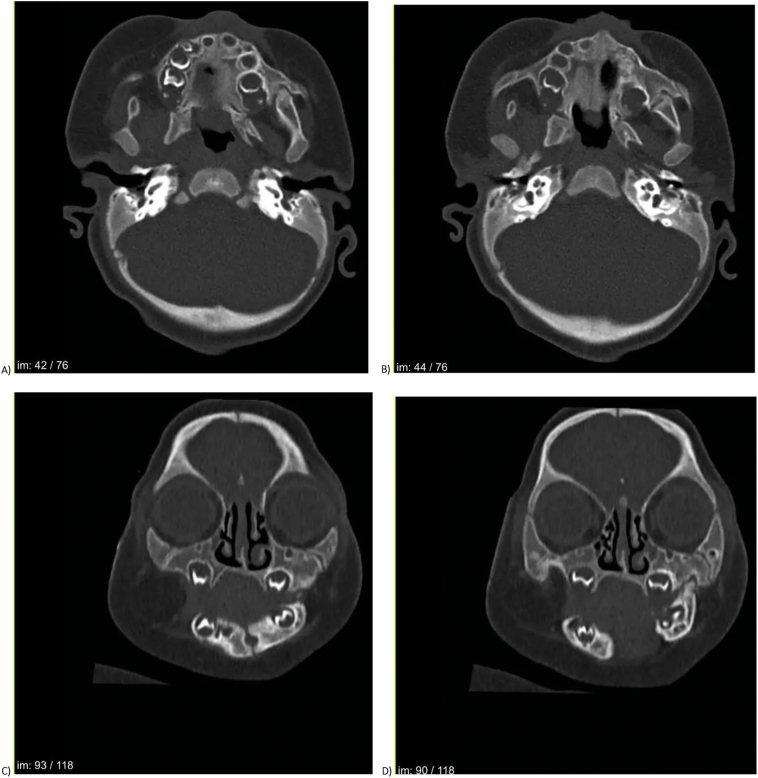
(A, B) Axial CT scan demonstrating bony fusion between the maxilla and mandible. (C, D) Coronal CT scan showing the extent of fusion at the alveolar level without involvement of the ramus or zygomatic arch.

Computed tomography (CT) of the craniofacial region revealed fusion of the maxilla and mandible at both the alveolar and bony levels, sparing the temporomandibular joints (Fig. 3). The differential diagnosis included syndromes associated with craniofacial abnormalities such as Nager syndrome and van der Woude syndrome. Genetic testing was initiated to investigate syndromic associations. The genetic analysis revealed a 1.66 Mb deletion at Xp22.31 (arr[GRCh37] Xp22.31 (6463468–8,120,430) × 1), encompassing genes such as *STS*, *VCX*, and *PNPLA4*.

## 5 Therapeutic interventions

**Fig. 4:**
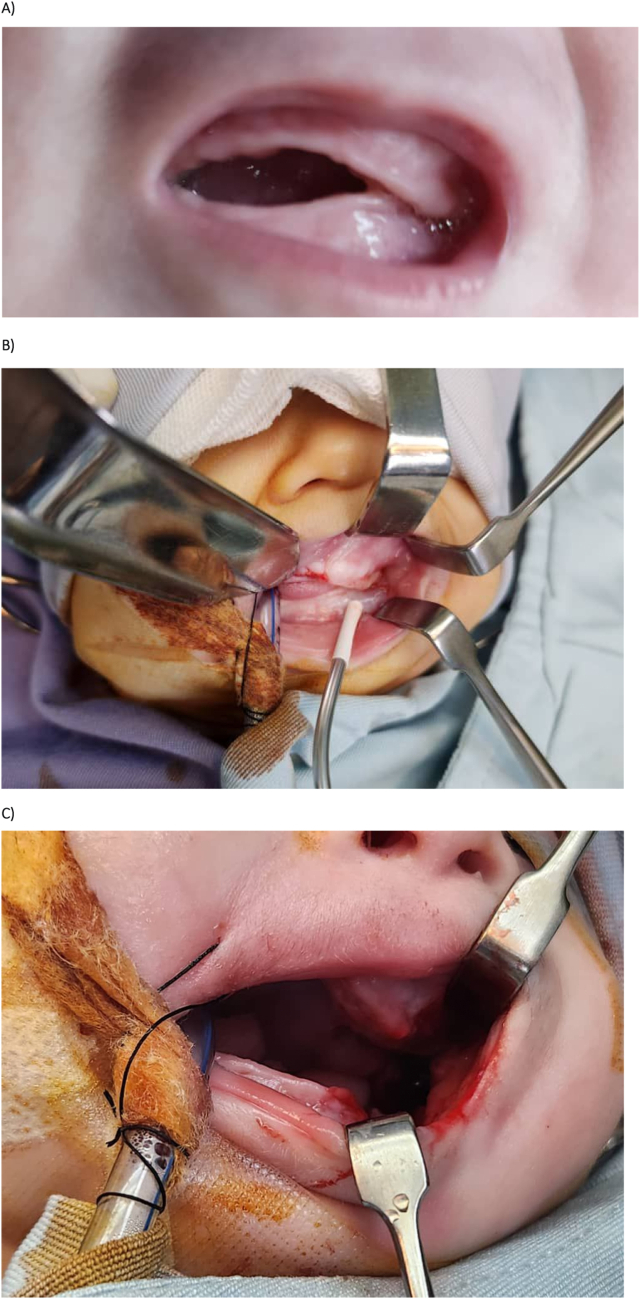
Surgical management of maxillomandibular fusion. (A) Intraoperative view showing the mucosal and bony fusion between the maxilla and mandible before separation. (B, C) Surgical separation of the fused maxilla and mandible, with meticulous dissection of both soft tissue and bony adhesions. (D, E) Placement of a fixation stent between the maxilla and mandible to prevent re-fusion, maintained for three days post-surgery.

The patient underwent surgical correction of syngnathia under general anesthesia. The mucosal and bony adhesions were separated to restore maxillomandibular mobility. A custom stent was placed between the maxilla and mandible to maintain the separation and prevent refusion during the healing process. The stent was removed on day three post-surgery. Postoperative care included monitoring for feeding difficulties and stent stability. Oral feeding was initiated following stent removal, with no signs of complications or refusion (Fig. 4).

## 6 Follow-up and outcomes

The patient had improved feeding and weight gain at follow-up two weeks after surgery. The craniofacial separation remained stable, with no signs of recurrence of syngnathia. Repeat echocardiography one month post-surgery showed no progression of cardiac anomalies, and the hemangioma on the right thigh remained stable. Further follow-up was planned for genetic evaluation and monitoring of neurodevelopmental problems. The prognosis was generally good, although ongoing developmental assessment and interdisciplinary management were necessary.

## 7 Discussion

We reported a 15-week-old female infant with congenital syngnathia, microcephaly, a right thigh hemangioma, and minor cardiac anomalies. Successful surgical separation of the bony and mucosal adhesions restored oral function, with no recurrence observed during short-term follow-up. This case highlights the critical role of early surgical intervention and multidisciplinary care in managing complex congenital anomalies such as syngnathia. The potential for underlying syndromic associations underscores the importance of genetic evaluation and long-term follow-up.

The coexistence of syngnathia with microcephaly, hemangioma, and cardiac defects suggests a broader developmental disorder. While syngnathia is often isolated, syndromic associations—such as Dobrow syndrome—typically include severe malformations (e.g., limb defects, coloboma) [10]. However, our patient had a milder phenotype, with preserved limb structure and no cleft palate, contrasts with cases like the case report by Broome et al., who reported multisystem anomalies [11]. The hemangioma and cardiac defects may indicate disrupted vascular or neural crest development as there was an association between BMP signaling dysregulation to craniofacial fusion [8].

The diagnosis of syngnathia in this case was established through clinical examination and CT imaging, which showed bony fusion at the alveolar level involvement. This is in accordance with the findings from Broome et al. [11], who utilized 3D CT to define fusion extent in syndromic syngnathia, and Mohan [7], who emphasized imaging to differentiate syngnathia from temporomandibular joint ankylosis. While prenatal ultrasound did not detect syngnathia in our case, its potential use for early diagnosis in case of presence of polyhydramnios or micrognathia were suggested [12].

We found a deletion at Xp22.31. It is typically associated with X-linked ichthyosis in males due to *STS* haploinsufficiency and it may contribute to the patient's clinical features through skewed X-inactivation. While heterozygous females are usually asymptomatic carriers, rare manifestations like corneal opacities and mild dermatologic findings have been reported [13]. The Xp22.31 deletion expands the phenotypic spectrum of this genomic region, which is not classically associated with craniofacial fusion. Syngnathia has been associated with neural crest dysregulation [14], but the absence of *STS* or *VCX* in such pathways implies a multifactorial etiology. The cardiac defects may reflect broader embryological disturbances rather than direct gene effects. This case underscores the complexity of genotype-phenotype correlations in syndromic presentations. Therefore, genetic counseling might be necessary to address recurrence risks and potential manifestations in offspring.

Surgical intervention involved mucosal and bony adhesion separation followed by stent placement to prevent refusion. Another study observed that better functional outcomes with prompt intervention [6]. The use of a custom stent is a technique of using mechanical barriers to counteract scar contracture [3]. Notably, the intact temporomandibular joint in our case simplified surgical access compared to cases with zygomatico-mandibular fusion, where extracoral approaches are often required [15]. The decision to remove the stent after three days in our case was based on the absence of refusion. As a result, it shows that shorter durations may suffice in limited fusion cases. The favorable surgical outcome underscores the importance of early intervention. Recurrence rates is high in extensive bony fusion, but stent use and postoperative physiotherapy can contribute to stability [3].

Postoperatively, our patient demonstrated restored oral mobility and successful oral feeding, consistent with outcomes reported by Hegab et al. [16]. The lack of recurrence at two-week follow-up can be due to the lower complexity of alveolar-level fusion. However, long-term follow-up should be considered, as delayed asymmetry or growth disturbances may occur. The stable cardiac defects (i.e., PDA/VSD) and hemangioma reflect the non-progressive nature of these anomalies in isolation, though syndromic cases often correlate with worsening multisystem involvement [11].

A strength of our approach was the early surgical intervention, which is in accordance with recommendations by Mohan et al., who emphasized prompt management to prevent feeding and respiratory complications [7]. A novel approach was the use of a custom stent postoperatively to maintain separation and reduce refusion risk. Multidisciplinary care involving neonatology, genetics, and maxillofacial surgery was an example of a comprehensive evaluation, which led to favorable outcomes. However, limitations include the absence of long-term follow-up data and lack of imaging studies during follow-up.

## 8 Conclusions

Our case report highlights the importance of early surgical correction and multidisciplinary care in managing syngnathia. While associated anomalies may suggest syndromic origins, genetic evaluation can be necessary in non-consanguineous families. The combination of microcephaly, hemangioma, and cardiac defects can be in favor of syngnathia-associated conditions. Custom stents and postoperative monitoring are effective strategies to prevent refusion. Clinicians should maintain a high index of suspicion for syndromic associations and prioritize long-term developmental follow-up to optimize outcomes.

## Ethical approval

Ethical approval was not applicable for this case, as the ethics committee does not mandate approval for reporting such individual cases.

## Sources of funding

The authors received no financial support for this research.

## Author contributions

Conceptualization and study design: RM, ZSM, and FM; drafting of the manuscript: RM, ZSM, SHM, and FM. All authors had a critical role in revision of the manuscript for important intellectual content. The final manuscript was read and approved by all authors.

## Conflict of interest statement

The authors declare that they have no conflict of interests.

## Guarantor

Rahele Mousavi.

## Research registration number

Not applicable.

## Consent

Written informed contest was received from the guardian of the patient.

## Data Availability

The data that support the findings of this study are available on request from the corresponding author. The data are not publicly available due to privacy or ethical restrictions.
